# Supplementary material for: Asymmetric dinitrogen-coordinated nickel single-atomic sites for efficient CO2 electroreduction
Source: Nat Commun. 2023 Jun 24;14:3776. doi: 10.1038/s41467-023-39505-2 (PMC10290683; doi:10.1038/s41467-023-39505-2)
Supplement: Supplementary file 1 — Supplementary information [file 41467_2023_39505_MOESM1_ESM.pdf]

Supplemental Information

Asymmetric Dinitrogen-Coordinated Nickel Single-Atomic Sites for Efficient CO_2 Electroreduction

Yuzhu Zhou,[1,4] Quan Zhou,[1,4] Hengjie Liu,[1,4] Wenjie Xu,[1] Zhouxin Wang,[1] Sicong Qiao,[1] Honghe Ding,[1] Dongliang Chen,[2] Junfa Zhu,[1] Zeming Qi,[1] Xiaojun Wu,[3] Qun He,[1,]* and Li Song[1,]*

[1]National Synchrotron Radiation Laboratory, CAS Center for Excellence in Nanoscience, University of Science and Technology of China, Hefei 230029, China.

[2]Beijing Synchrotron Radiation Facility, Institute of High Energy Physics, Chinese Academy of Sciences, Beijing 100049, China.

[3]Hefei National Laboratory for Physical Science at the Microscale, Collaborative Innovation of Center of Chemistry for Energy Materials (iChEM), School of Chemistry and Materials Sciences, University of Science and Technology of China, Hefei 230026, China.

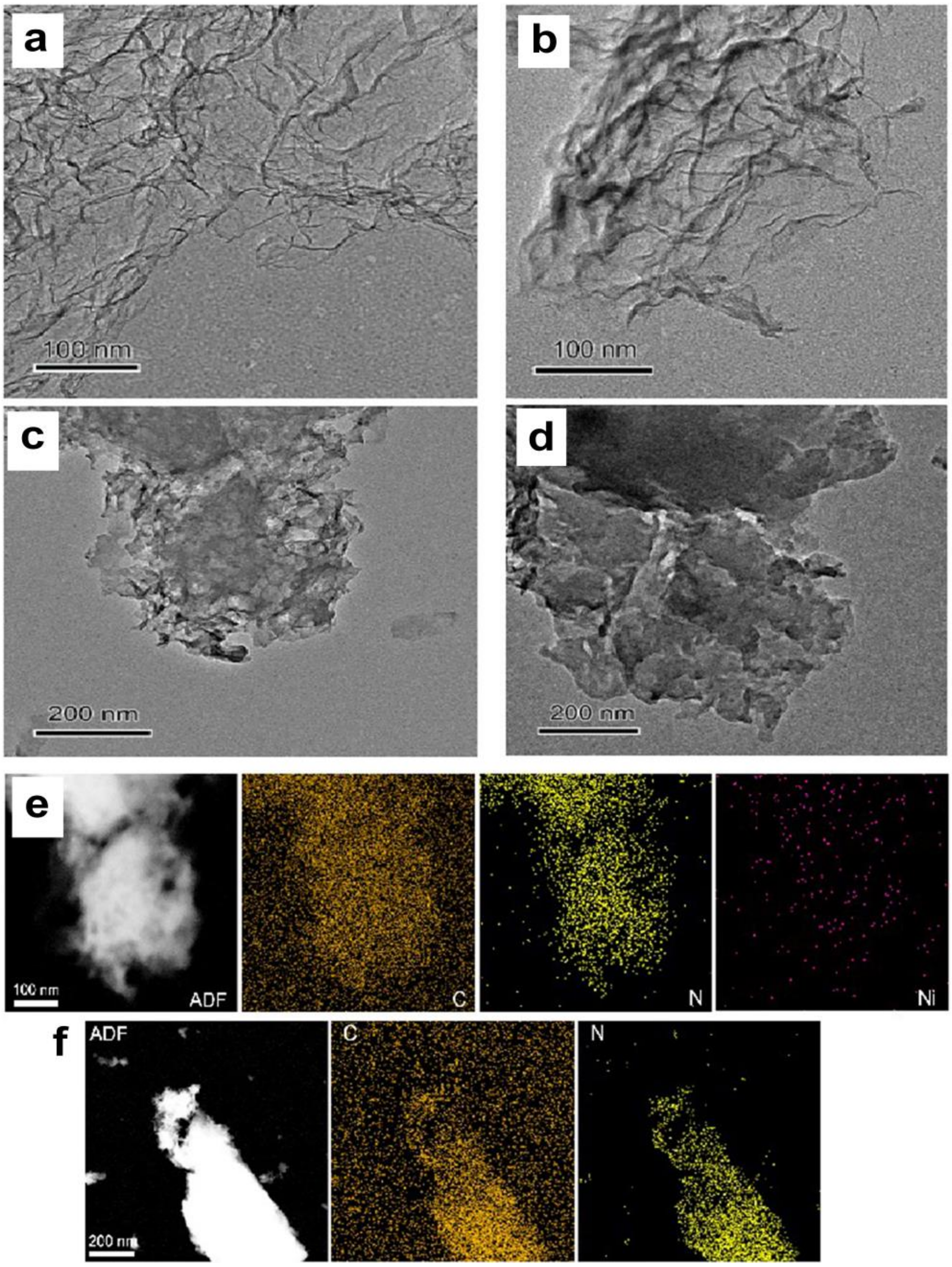

Fig. S1. TEM images of (**a**) Ni-N-C, (**b**) NC, (**c**) Ni-N-C precursor, and (**d**) NC precursor. Elemental mappings of (**e**) Ni-N-C precursor and (**f**) NC precursor.

We first carried out TEM analysis to analyze the structural changes of g-C_3N_4 with and without the metal. TEM observation reveals a typical stacking structure for these samples (fig. S1a,b). Previous work corroborated that DICY was first assembled to form laid graphic carbon nitrate (g-C_3N_4) at low temperature, which served as a template to confirm and guide patches of aromatic carbon intermediate (derived from the calcination of glucose) condensation between the interlayer gaps[1]. Elemental mapping analysis reveals the uniform distribution of g-C_3N_4 in both precursors (fig. S1e,f). For Ni-N-C and NC, TEM observation reveals a uniform, wrinkled sheet-like structure (fig. S1c,d).

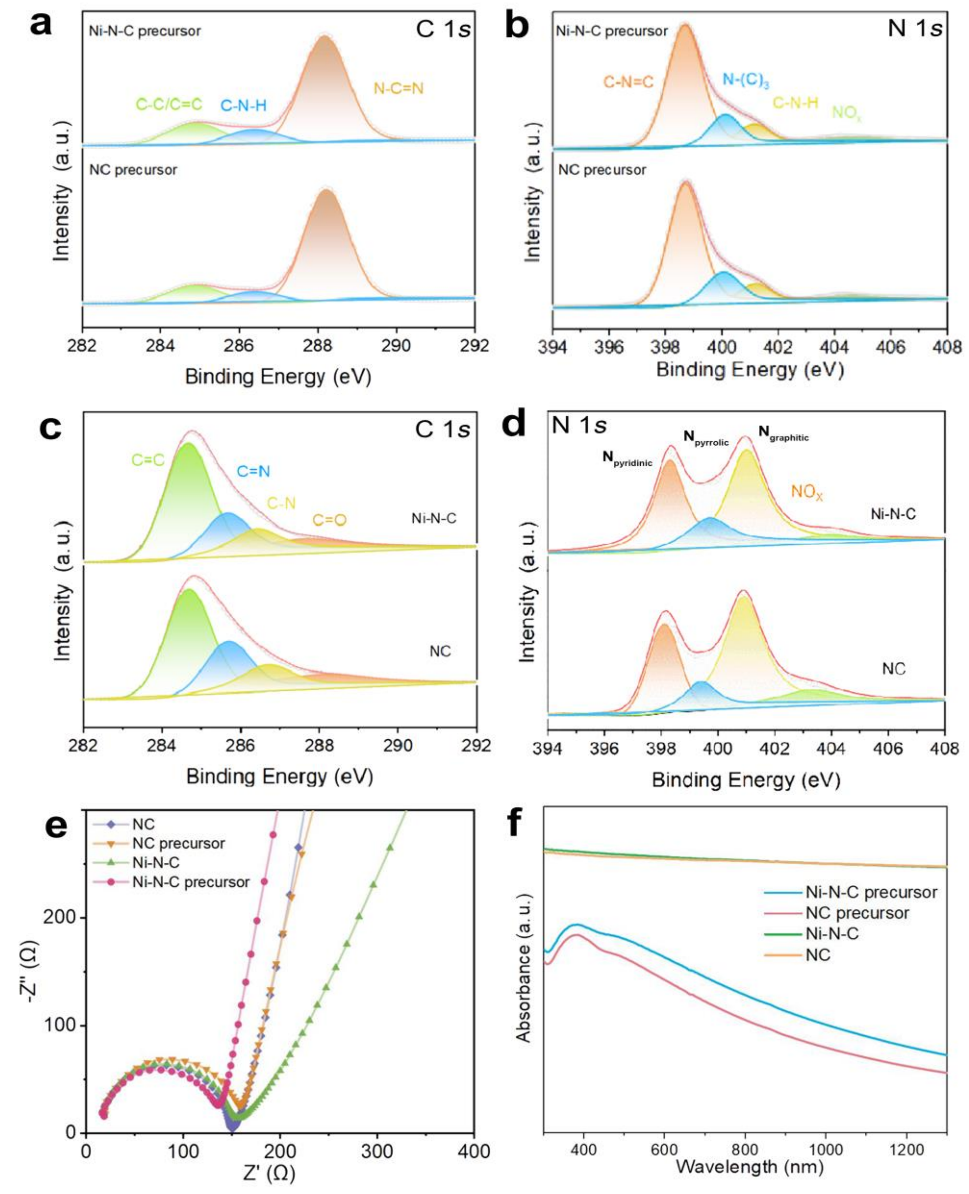

Fig. S2. XPS spectra of C1*s* (**a**) and N 1*s* (**b**) for precursors of NC and Ni-N-C. XPS spectra of C1s (**c**) and N 1*s* (**d**) for NC and Ni-N-C. (**e**) Nyquist plots of NC, Ni-N-C, and their precursors measured at OCP in 0.5 M $KHCO_3$. (**f**) UV-Vis-NIR spectra of various samples.

XPS analysis also reveals the presence of g-C_3N_4 in both precursors (fig. S2a,b). The C atoms are primarily *sp*^{2} type, indicating that the precursors have transformed into a N-doped graphene structure at high temperatures (fig. S2c). This is consistent with our previous work, which shows that the g-C_3N_4 can be completely decomposed at 750 °C[2]. Furthermore, N 1*s* XPS spectra of both Ni-N-C and NC show that they mainly contain graphitic, pyridinic, and pyrrolic N, further evidence the complete transformation of precursors (fig. S2d). Additionally, it is found that compared with NC, both pyridinic and pyrrolic N peaks of Ni-N-C show a slight shift to higher energies, which indicate the partial electron transfer from N to Ni. There results clearly demonstrated the structural changes of the g-C_3N_4 and treated NC with and without the metal.

Based on Nyquist plots analysis, it can be observed that all samples show comparable charge transfer resistance (Fig. S2e). This can be attributed to the formation of an electrically conducting carbon layer resulting from glucose carbonization between the g-C_3N_4 structure, which is further supported by UV-Vis results (Fig. S2f)[1]. Combined with the structure analysis, it can be concluded that the structure and electrical properties of N-doped carbon supports are analogous and not the main factors influencing the performance. The performance is primarily derived from the Ni single-atom sites.

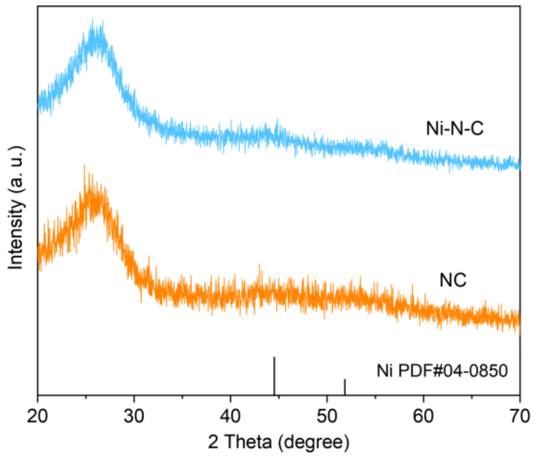

Fig. S3. XRD patterns of Ni-N-C and NC.

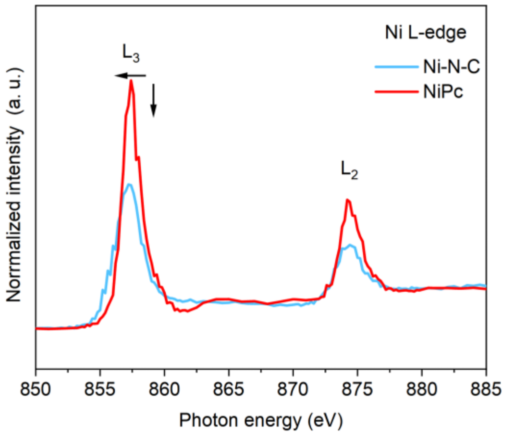

Fig. S4. Ni L-edge XANES spectra of Ni-N-C and NiPc.

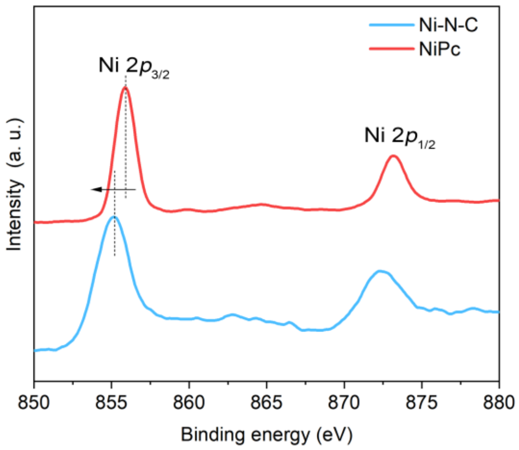

Fig. S5. Ni 2*p* XPS spectra of Ni-N-C and NiPc.

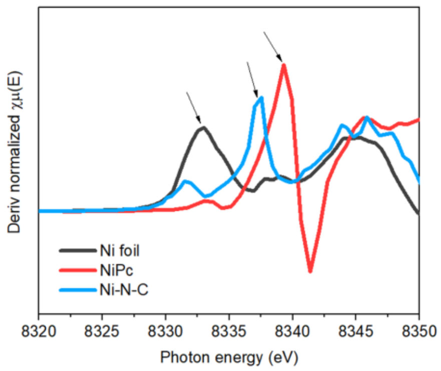

Fig. S6. 1st derivative data of Ni K-edge XANES for Ni foil, Ni-N-C, and NiPc.

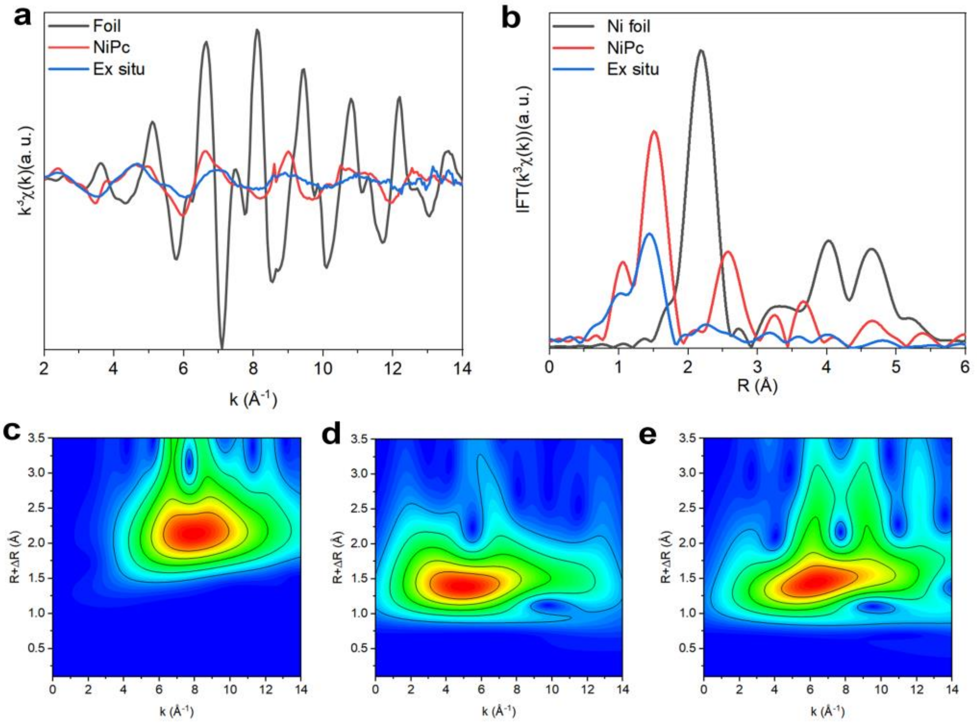

Fig. S7. a The oscillation curves of Ni-N-C, NiPc and Ni foil. **b** EXAFS spectra of Ni-N-C, NiPc and Ni foil. **c-e** Wavelet transform of Ni foil, Ni-N-C and NiPc, respectively.

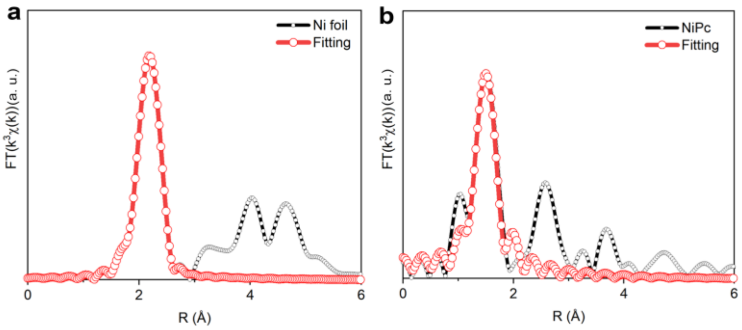

Fig. S8. EXAFS fitting curves of (**a**) Ni foil and (**b**) NiPc.

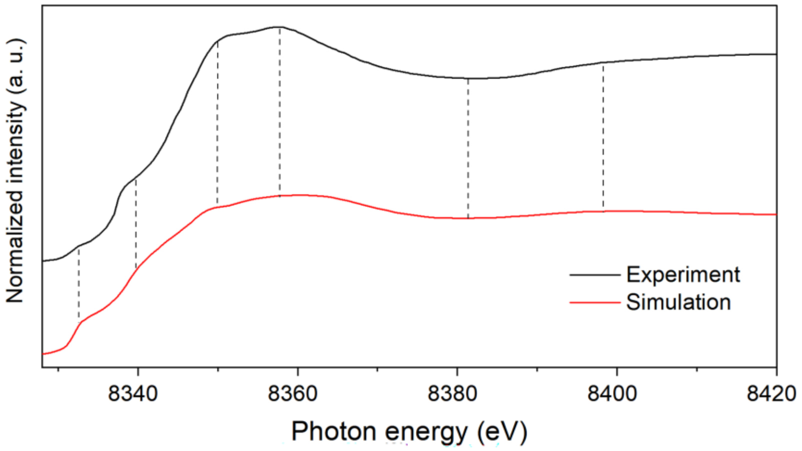

Fig. S9. Comparison between the experimental XANES spectrum of Ni and the simulated spectrum.

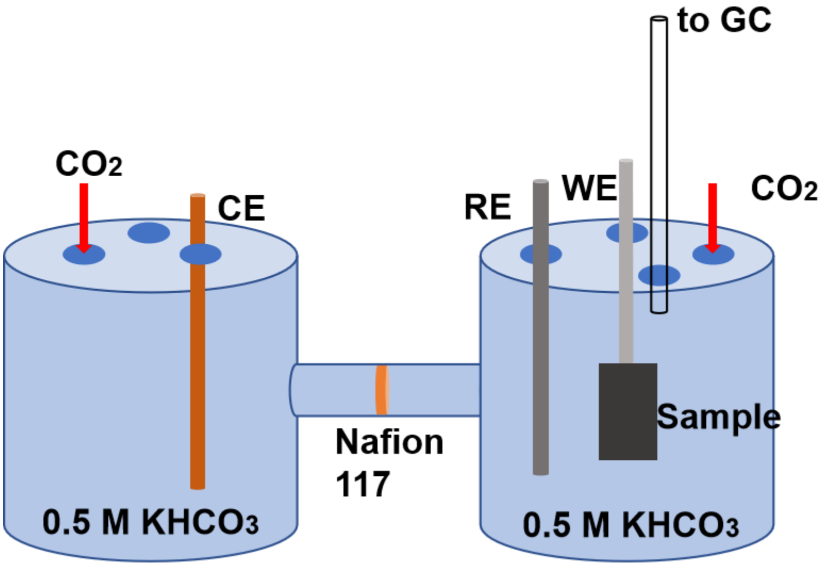

Fig. S10. Schematic illustration of H-type cell test.

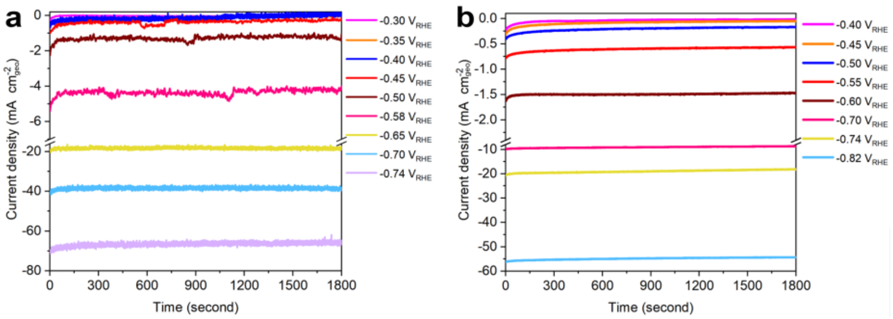

Fig. S11. Chronoamperometry curves of (**a**) Ni-N-C and (**b**) NiPc measured at applied potentials in CO_2-saturated 0.5 M $KHCO_3$.

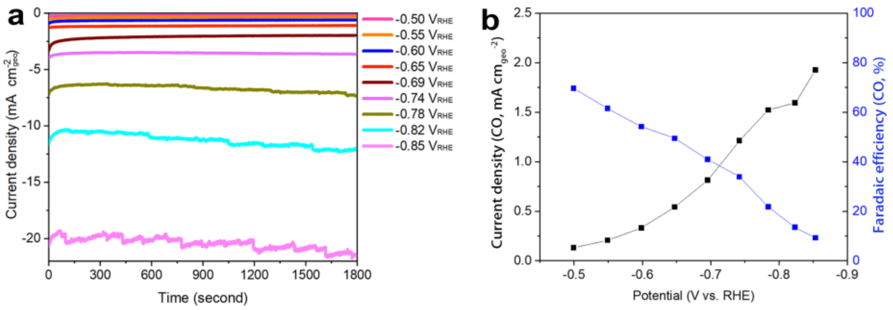

Fig. S12. Chronoamperometry curves (**a**) and FE_{CO}/j_{CO} (**b**) of NC measured at applied potentials in CO_2-saturated 0.5 M $KHCO_3$.

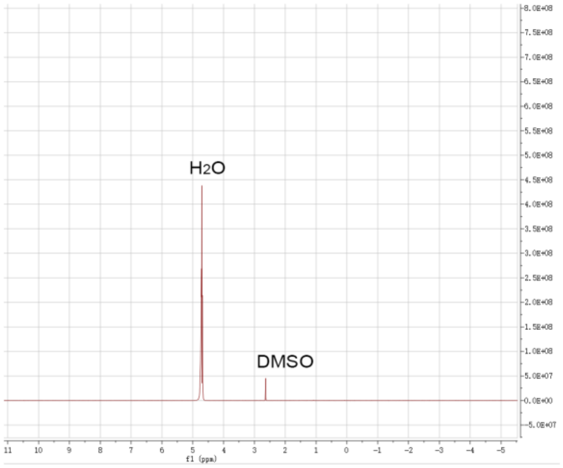

Fig. S13. A typical ^{1}H NMR spectrum of Ni-N-C measured at -0.5 V_{RHE}.

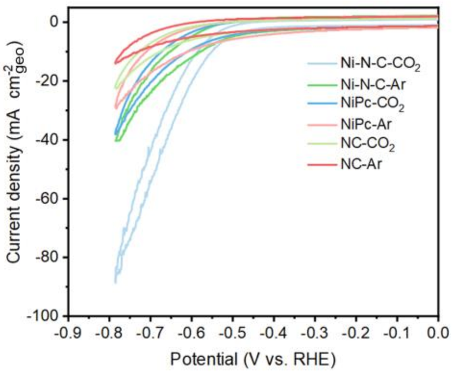

Fig. S14. CV curves of Ni-N-C, NiPc, and NC in Ar and CO_2 saturated 0.5 M $KHCO_3$ at a scan rate of 50 mV s^{-1}.

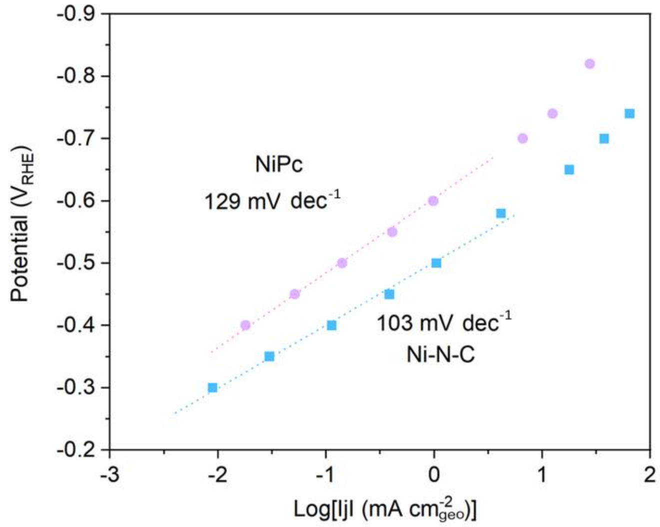

Fig. S15. Tafel slopes of Ni-N-C and NiPc.

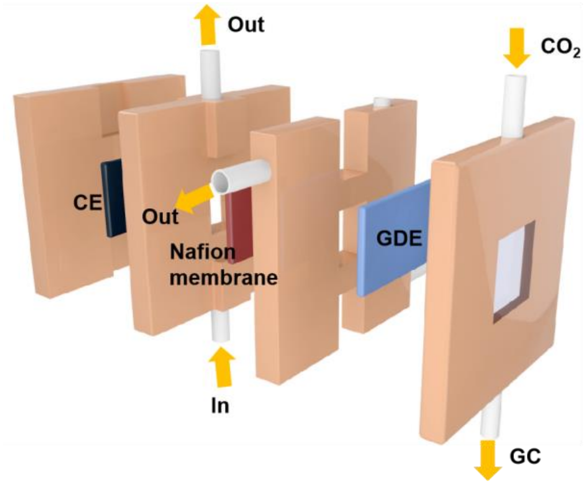

Fig. S16. Schematic illustration of flow cell test.

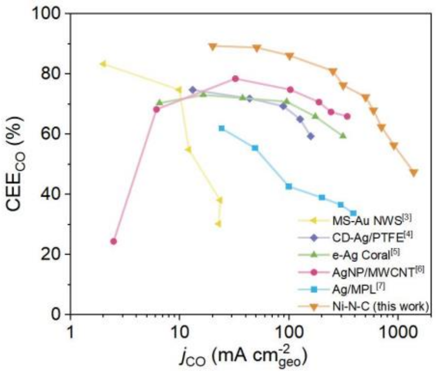

Fig. S17. CEE comparison of Ni-N-C with reported catalysts.

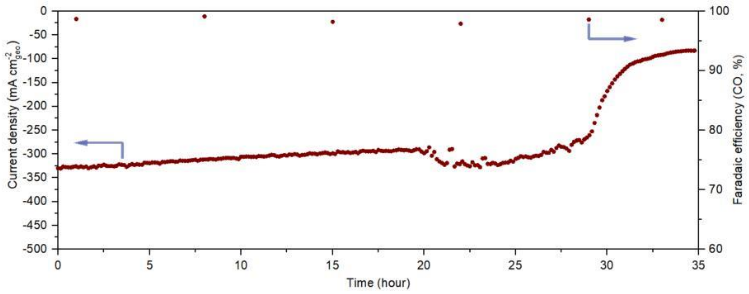

Fig. S18. Chronoamperometry curve and FE_{CO} of Ni-N-C measured at -0.5 V_{RHE} for over 30 hours in GDE with 1.0 M KOH as electrolyte.

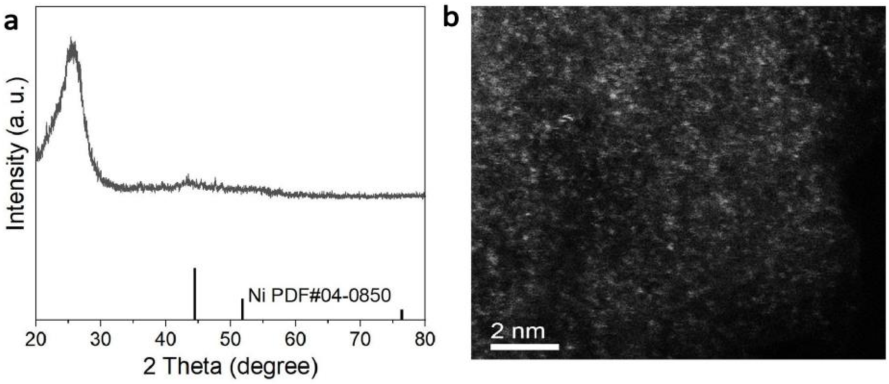

Fig. S19. Structural analysis of Ni-N-C after operation. (**a**) XRD and (**b**) HAADF-STEM analysis of Ni-N-C after operation.

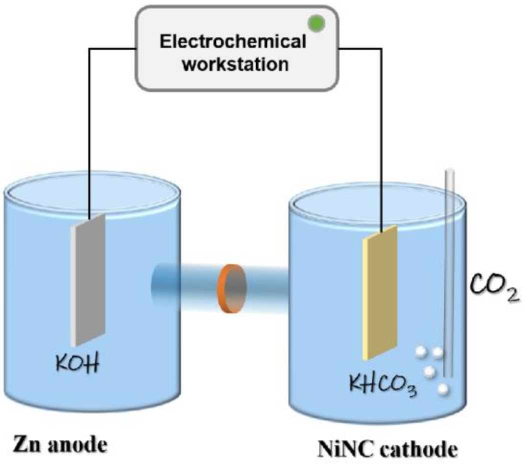

Fig. S20. Schematic illustration of Zn-CO_2 battery test.

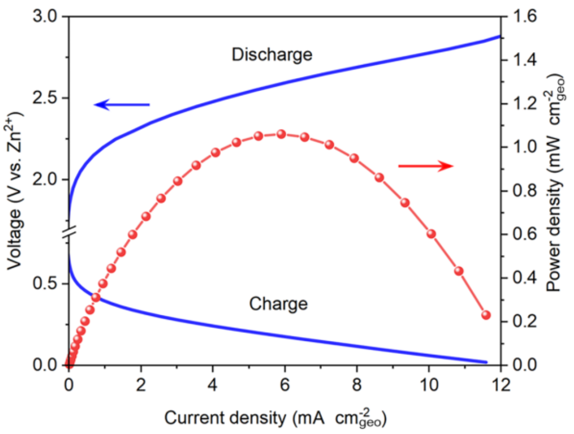

Fig. S21. Charge and discharge polarization curves, together with the power density during discharge process for Ni-N-C.

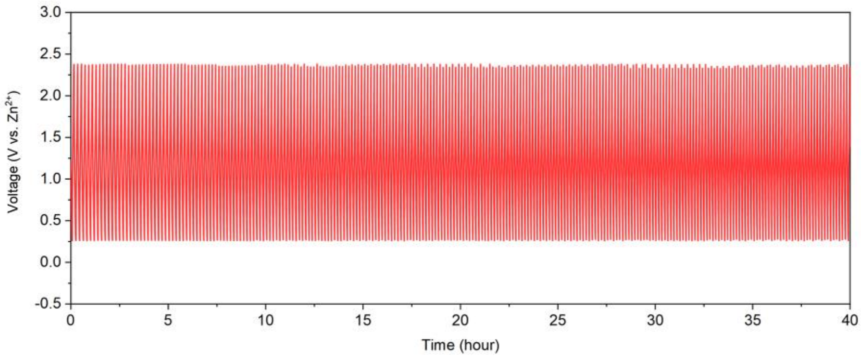

Fig. S22. Galvanostatic discharge-charge cycling curves of Ni-N-C measured at 2.0 mA cm_{geo}^{-2}.

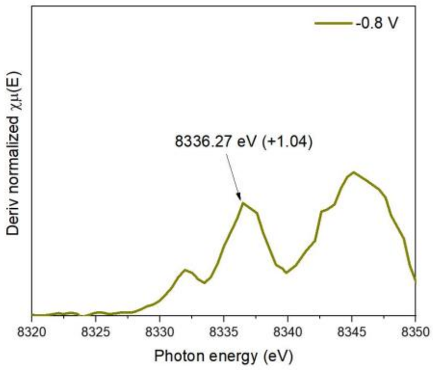

Fig. S23. 1st derivative data of Ni K-edge XANES for Ni-N-C at -0.8 V_{RHE}.

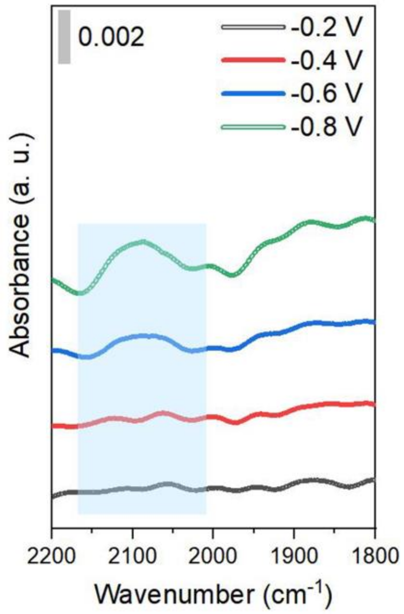

Fig. S24. In situ SR-IRAS spectra of NiPc collected in CO_2-saturated 0.5 M $KHCO_3$.

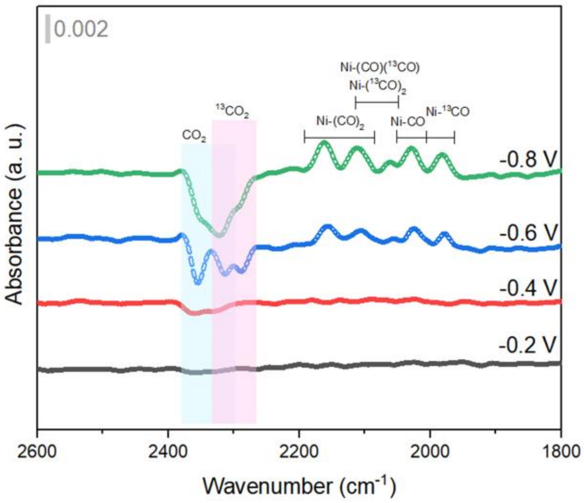

Fig. S25. In situ isotope analysis for Ni-N-C in $^{13}CO_2$-purged 0.5M $KHCO_3$.

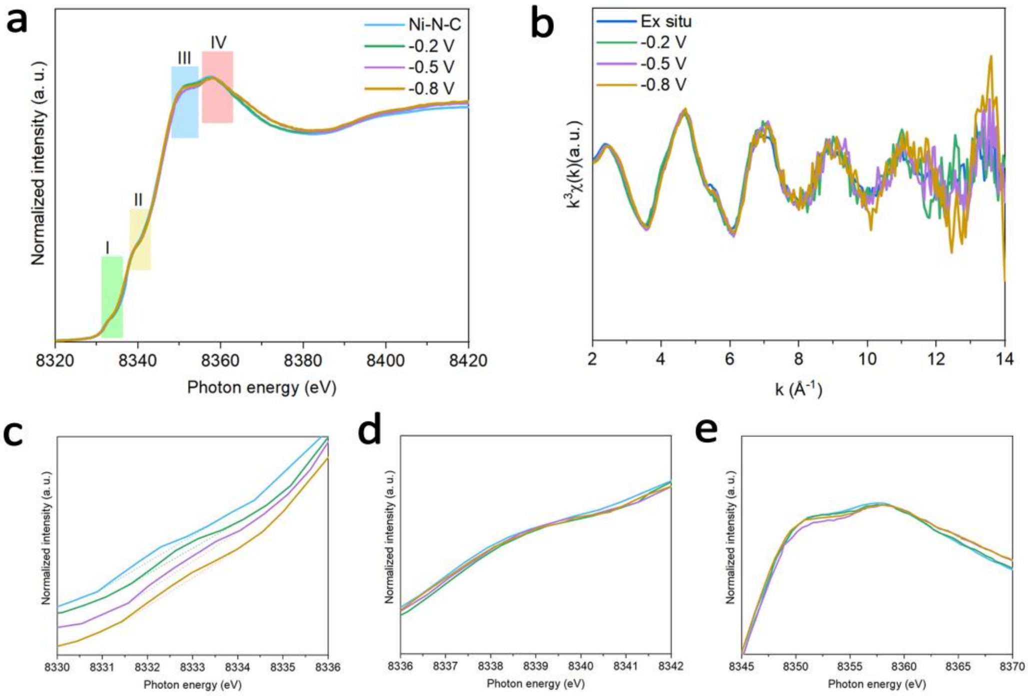

Fig. S26. Operando XAFS analysis under various potentials for Ni-N-C in CO_2-saturated 0.5 M $KHCO_3$. (**a**) Ni K-edge XANES spectra of Ni-N-C collected at various potentials, (**b**) corresponding oscillation curves. (**c**-**e**) Zoomed-in details of features I-IV in **a**.

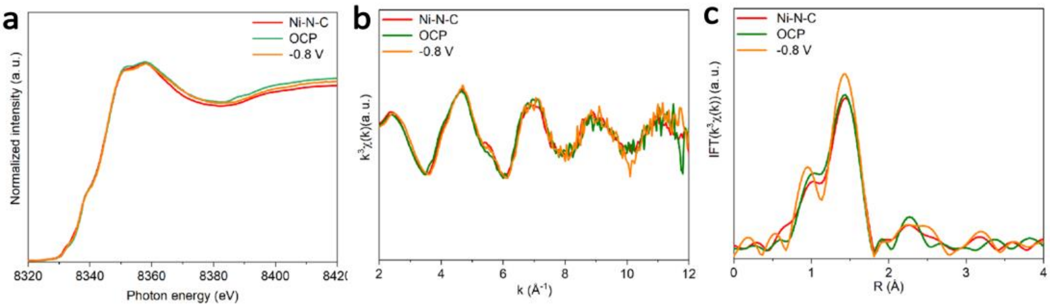

Fig. S27. Ex situ and operando XAFS analysis under various potentials for Ni-N-C. (**a**) Ni K-edge XANES spectra of Ni-N-C collected at OCP after electrolysis, together with ex situ and -0.8 V_{RHE} spectra, (**b**) corresponding oscillation curves and (**c**) FT-EXAFS spectra.

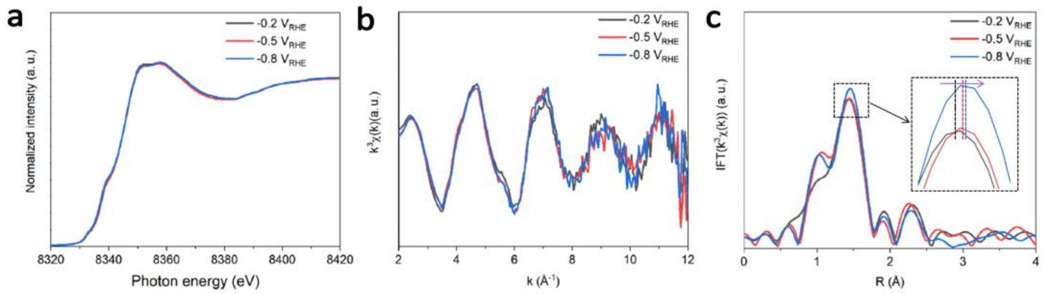

Fig. S28. Operando XAFS analysis under various potentials for Ni-N-C in Ar-saturated 0.5 M $KHCO_3$. (**a**) Ni K-edge XANES spectra of Ni-N-C collected at various potentials, (**b**) corresponding oscillation curves and (**c**) FT-EXAFS spectra.

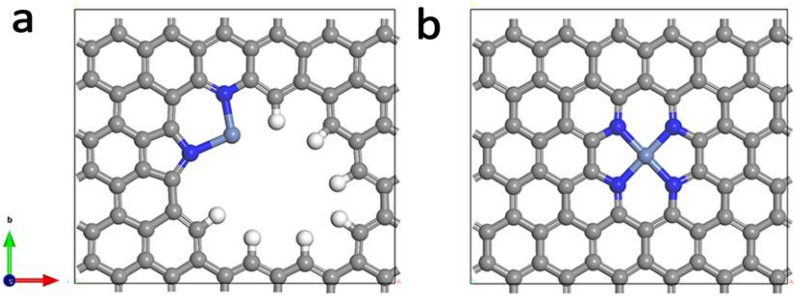

Fig. S29. The structures of NiN_2 (**a**) and NiN_4 (**b**).

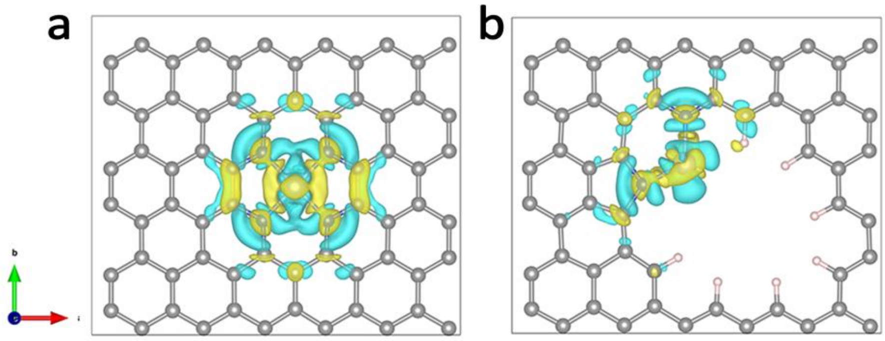

Fig. S30. Differential charge density analysis of NiN_4 (**a**) and NiN_2 (**b**).

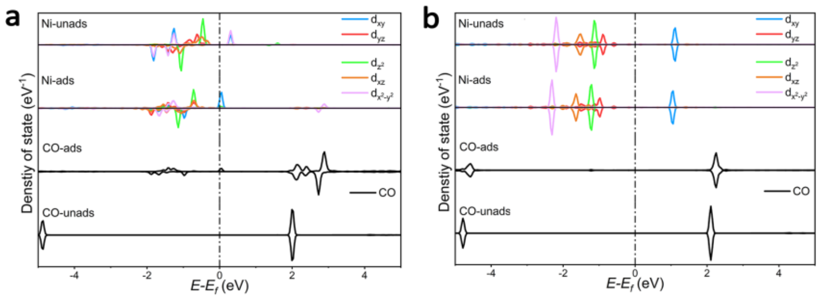

Fig. S31. Projected density of states of NiN_2 (**a**) and NiN_4 (**b**) before and after CO adsorption.

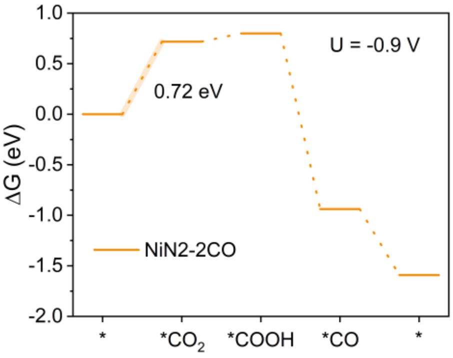

Fig. S32. DFT calculated reaction free energy diagrams of NiN_2-2CO for CO_2RR.

Table S1. ICP-AES analysis to evaluate the Ni loading in Ni-N-C and NiPc.

Sample	Ni-N-C	NiPc
Mass ratio of Ni (%)	1.10	1.51

Table S2. ICP-AES analysis to evaluate the Ni loading in Ni-N-C and NiPc.

Sample	Ni foil	Ni-N-C	NiPc
Edge energy (eV)	8333.00	8337.30	8339.28
Valence states	0.00	+1.37	+2.00

Table S3. EXAFS fitting data of Ni-N-C, Ni foil and NiPc.

Sample	Path[#1]	N[#2]	R (Å)[#3]	σ^2 (10^{-3} Å^2)[#4]	abs(ΔE_0) (eV)[#5]	R factor[#6]
Ni-N-C	Ni-N	2.2	1.86	6.11	9.13	0.017
Ni foil	Ni-Ni	12.0	2.48	6.57	6.60	0.003
NiPc	Ni-N	4.0	1.88	4.69	2.40	0.014

[#1]Scattering paths

[#2]Coordination number

[#3]Bond length

[#4]Debye-Waller factor

[#5]Energy shift

[#6]Degree of curve coincidence

Table S4. Activity comparison of Ni-N-C with recently reported catalysts at -0.7 V_{RHE} (H cell).

Catalysts	Electrolyte	FE_{CO} (%)	j_{CO} (mA cm_{geo}^{-2})	Reference
Ni-N-C	0.5 M $KHCO_3$	98.5	37.6	this work
Ni-CNC-1000	0.5 M $KHCO_3$	95.0	3.2	(*8*)
N_3NiPc-CNT	0.5 M $KHCO_3$	99.0	13.5	(*9*)
Ni-N_4/C-NH_2	0.5 M $KHCO_3$	92.0	30.8	(*10*)
A-Ni-NSG	0.5 M $KHCO_3$	96.5	26.5	(*11*)
NiFe-DASC	0.5 M $KHCO_3$	93.5	20.7	(*12*)
C-Zn_1Ni_4 ZIF-8	0.5 M $KHCO_3$	98.0	40.0	(*13*)
CoN_4-CNT	0.5 M $KHCO_3$	95.5	40.6	(*14*)
Fe_2-N_6-C-o	0.5 M $KHCO_3$	89.0	11.9	(*15*)
Co-N_2	0.5 M $KHCO_3$	89.5	22.4	(*16*)
CoTMAPc@CNT	0.5 M $KHCO_3$	98.5	19.2	(*17*)
Ag-200nm NWA	0.5 M $KHCO_3$	84.0	10.3	(*18*)
Au sputtered electrode	0.5 M $KHCO_3$	70.0	3.3	(*19*)

Table S5. Comparison of applied potentials to achieve a j_{CO} of about 20 mA cm_{geo}^{-2} in flow cell.

Catalysts	Electrolyte	Potential (V_{RHE})	Reference
Ni-N-C	1.0 M KOH	~-0.15	this work
Ni-CNC-1000	1.0 M KOH	~-0.32	(*8*)
N_3NiPc-CNT	1.0 M KOH	~-0.31	(*9*)
Ni-N_4/C-NH_2	1.0 M KOH	~-0.30	(*10*)
CoPc2	1.0 M KOH	~-0.31	(*20*)

Table S6. EXAFS fitting data of Ni-N-C measured at various potentials.

Sample	Path	N	R (Å)	σ^2 (10^{-3} Å^2)	abs(ΔE_0) (eV)	R factor
Ex situ	Ni-N	2.20	1.86	6.11	9.13	0.017
-0.2 V_{RHE}	Ni-N(O)	2.40	1.88	3.63	4.59	0.008
-0.5 V_{RHE}	Ni-N(C)	2.45	1.85	4.13	7.94	0.009
-0.8 V_{RHE}	Ni-N(C)	2.56	1.85	3.71	5.73	0.011

Table S7. Theoretical bond lengths in our models.

Adsorption species	None	H_2O	CO
Ni-N length	1.93	1.93	2.02
Ni-C/O length	/	2.06(Ni-O)	1.79(Ni-C)

Table S8. Bader charge of Ni atom in NiN_2 and NiN_4.

Slab	Bader charge (electron)
NiN_2	+0.608
NiN_4	+0.885

Supplementary references

1. Li, X.-H. et al. Synthesis of monolayer-patched graphene from glucose. *Angew. Chem. Int. Ed.* **51**, 9689-9692 (2012).

2. Liu, D. et al. In situ trapped high-density single metal atoms within graphene. *Nano Res.* **11**, *2217-2228* (2018).

3. Lee, W. H. et al. Highly selective and scalable CO_2 to CO-Electrolysis using coral-nanostructured Ag catalysts in zero-gap configuration. *Nano Energy* **76**, *105030* (2020).

4. Ma, S, C, et al. Carbon nanotube containing Ag catalyst layers for efficient and selective reduction of carbon dioxide. *J. Mate. Chem. A* **4**, *8573-8578* (2016).

5. Wang, R. M. et al. Maximizing Ag utilization in high-rate CO_2 electrochemical reduction with a coordination polymer-mediated gas diffusion electrode. *ACS Energy Lett.* **4**, *2024-2031* (2019).

6. Cho, M. et al. Versatile, transferrable 3-dimensionally nanofabricated Au catalysts with high-index crystal planes for highly efficient and robust electrochemical CO_2 reduction. *J. Mater. Chem. A* **7**, *6045-6052* (2019).

7. Dinh, C. T. et al. Selective, and stable electroreduction of CO_2 to CO in basic and neutral media. *ACS Energy Lett.* **3**, *2835-2840* (2018).

8. Cao, X. et al. Atomic bridging structure of nickel-nitrogen-carbon for highly efficient electrocatalytic reduction of CO_2. *Angew. Chem. Int. Ed.* **61**, e202113918 (2022).

9. Ma, D.-D. et al. Bifunctional single-molecular heterojunction enables completely selective CO_2-to-CO conversion integrated with oxidative 3D nano-polymerization. *Energy Environ. Sci.* **14**, 1544-1552 (2021).

10. Chen, Z. et al. Amination strategy to boost the CO_2 electroreduction current density of M-N/C single-atom catalysts to the industrial application level. *Energy Environ. Sci.* **14**, 2349-2356 (2021).

11. Yang, H. B. et al. Atomically dispersed Ni(I) as the active site for electrochemical CO_2 reduction. *Nat. Energy* **3**, 140-147 (2018).

12. Zeng, Z. et al. Orbital coupling of hetero-diatomic nickel-iron site for bifunctional electrocatalysis of CO_2 reduction and oxygen evolution. *Nat. Commun.* **12**, 4088 (2021).

13. Yan, C. et al. Coordinatively unsaturated nickel-nitrogen sites towards selective and high-rate CO_2 electroreduction. *Energy Environ. Sci.* **11**, 1204-1210 (2018).

14. Wang, C. et al. Diminishing the uncoordinated N species in Co-N-C catalysts toward highly efficient electrochemical CO_2 reduction. *ACS Catal.* **12**, 2513−2521 (2022).

15. Wang, Y. et al. Precisely constructing orbital coupling modulated dual-atom Fe pair sites for synergistic CO_2 electroreduction. *ACS Energy Lett.* **7**, 640-649 (2022).

16. Wang, X. et al. Regulation of coordination number over single Co sites: Triggering the efficient electroreduction of CO_2. *Angew. Chem. Int. Ed.* **57**, 1944-1948 (2018).

17. Su, J. et al. Building a stable cationic molecule/electrode interface for highly effiffifficient and durable CO_2 reduction at an industrially relevant current. *Energy Environ. Sci.* **14**, 483-492 (2021).

18. Luan, C. et al. High-performance carbon dioxide electrocatalytic reduction by easily fabricated large-scale silver nanowire arrays. *ACS Appl. Mater. Interfaces* **10**, *17950-17956* (2018).
19. Hong, S. et al. Anion dependent CO/H_2 production ratio from CO_2 reduction on Au electrocatalyst. *Cataly. Today* **295**, *82-88* (2017).
20. Wang, M. et al. CO_2 electrochemical catalytic reduction with a highly active cobalt phthalocyanine. *Nat. Commun.* **10**, 3602 (2019).

Supplementary Note

To calculate the TOF of CO formation in literature, we used the formula:

$$TOF_{CO} = I_{CO}/(2F*n_{site})$$

Where I_{CO} is partial current for CO production; F is the Faraday constant (96485.3 C mol^{-1}); n_{site} is the number of active sites.

For atomically dispersed catalysts, each metal site is considered as a catalytic site, while for bulk metal catalysts, the number of active sites is determined by electrochemical methods.

The details of n_{site} evaluation in the cited literature are introduced below.

(1) Zn_1Ni_4-ZIF-8 (*Energy Environ. Sci.* **11**, 1204-1210 (2018)): TOF data was extracted from Fig. 3d.

(2) Fe^{3+}-N-C (*Science* **364**, 1091-1094 (2019)): TOF data was extracted from Fig. 2d.

(3) OD-Au (*J. Am. Chem. Soc.* **134**, 19969-19972 (2012)): The electrochemical surface area of the oxide-derived Au electrode was determined by measuring the charge associated with the stripping of an underpotential deposited Cu monolayer (Fig. S4). It is assumed that the atomic density on the electrochemical surface is that on Au (111) facet and all surface atoms were active sites.

(4) np-Ag (*Nat. Commun.* **5**, 3242 (2014)). The CO partial current density was determined using Fig. 7 in Supplementary Materials, and the electrochemical surface area for per 1 cm^2 of the electrode was calculated as 2650 cm^2 using cyclic voltammetry (Fig. 8 in Supplementary Materials). TOF was determined by assuming Ag (111) facet as the active site.

(5) CoPc-2 (*Nat. Commun.* **10**, 3602 (2019)): The CO partial current density was extracted from Fig. 5a, and active site concentration was obtained from Co^{II}/Co^{I} redox wave from CV under argon atmosphere (Fig. 2).

(6) A-Ni-NSG (*Nat. Energy* **3**, 140-147 (2018)): TOF data was extracted from Fig. 3c.

(7) $NiN_4/C-NH_2$ (*Energy Environ. Sci.* **14**, 2349-2356 (2021)): The CO partial current densities in H-cell and flow cell were extracted from Fig. 2c and Fig. 3e, respectively. They assumed that the metals on the electrodes were atomically dispersed, and each site was regulated as a catalytic site. Active site concentration was estimated from catalyst loading versus metal concentration.

(8) Ni-CNC-1000 (*Angew. Chem. Int. Ed.* **61**, e202113918 (2022)): TOF data in H-cell was extracted from Fig. 3e, while the Faradaic efficiency and CO partial current density in the flow cell were extracted from Fig. 5c. They assumed that the metals on the electrodes were atomically dispersed, and each site was regulated as a catalytic site. Active site concentration was estimated from catalyst loading versus metal concentration.

(9) NiFe-DASC (*Nat. Commun.* **12**, 4088 (2021)): The CO partial current density was extracted from Fig. 3d. Assuming that the Fe-Ni atom pair is catalytic site, and site concentration was estimated from catalyst loading versus metal concentration on the electrodes.

(10) N_3NiPc-CNT (*Energy Environ. Sci.* **14**, 1544-1552 (2021)): TOF data in H-cell was extracted from Fig. 3d, and the Faradaic efficiency of CO and total current densities in the flow cell were extracted from Fig. 3h. Active site concentration was estimated from catalyst loading versus metal concentration on the GDE.

(11) CoN_4-CNT (*ACS Catal.* **12**, 2513-2521 (2022)): TOF data was extracted from Fig. 3e.
